# Ion and Water Absorption by the Kidney Proximal Tubule: Computational Analysis of Isosmotic Transport

**DOI:** 10.1093/function/zqaa014

**Published:** 2020-08-27

**Authors:** Erik H Larsen, Jens N Sørensen

**Affiliations:** 1 Department of Biology, University of Copenhagen, Copenhagen, Denmark; 2 Department of Wind Energy, Technical University of Denmark, Lyngby, Denmark

The major function of the kidney’s proximal convoluted tubules is the reabsorption of filtered water and sodium, a transport largely mediated by conductive Na^+^ and hydraulic water pathways.^1^^,^^2^ Transepithelial water absorption is accomplished by isosmotic transport, the mechanism of which has been the subject of intense research and controversy. Here we briefly review a model of considerable explanatory power, which for the first time reveals what we believe is the precise mechanism by which basolateral sodium recirculation generates true isosmotic transport at an insignificant metabolic cost.^3^ Our results open the way for testing the predictions of the model in future experimental work. The governing electrodiffusion equations in the model include current equations for the rheogenic Na^+^/K^+^ pump and conductance equations for the luminal rheogenic sodium-glucose transporter SGLT2. The computed membrane resistances combined with electrical bridge circuit analysis provide values for the transepithelial resistance and the resistance of the luminal membrane relative to the transcellular resistance.^4^ The paracellular solute fluxes are computed by our integrated electrodiffusion–convection equation.^4^ The model produces values for the intracellular ion concentrations, cell volume, membrane potentials, and conductances that are in agreement with experimental results. The ionic composition, hydrostatic pressure, and volume of the lateral intercellular space (*lis*), inaccessible to measurement in the transporting tubule, can be obtained under any physiological condition.

In glucose-free solutions, with physiological Na^+^- and water fluxes of ∼5200 pmol/(cm^2^s) and 34 nL/(cm^2^s), respectively (Figure 1A), the shunt resistance of $5.5\;\Omega \cdot {\text{cm}}^{2}$ and the luminal membrane’s resistance of ∼$300\;\Omega \cdot {\text{cm}}^{2}$with an associated ratio of the resistance of the luminal membrane relative to the transcellular resistance of 0.76, are in good agreement with data from the rat.^1^ It is of particular interest that the osmolarity of the *lis* (305 mosM) is no more than 1.7% hyperosmotic relative to the bathing solutions (300 mosM), while the fluid exiting *lis* is 5.3% hyperosmotic (315 mosM). These numbers illustrate the fundamental convection–diffusion problem of isosmotic transport, ie, the relatively large diffusion coefficients of the interspace basement membrane governing exit fluxes from *lis*, cause the osmolarity of the absorbed fluid to become significantly higher than that of *lis*. Addition of 5 mM bilateral glucose induces a transepithelial hyperpolarization of −0.45 mV (from −1.78 to −2.23 mV) as compared to −0.36±0.22, mean±SD in the rat.^5^ When engaged in glucose absorption, the osmolarity of the emerging fluid is 335 mosM, thus deviating even more from isosmotic transport.^3^

**Figure 1.**
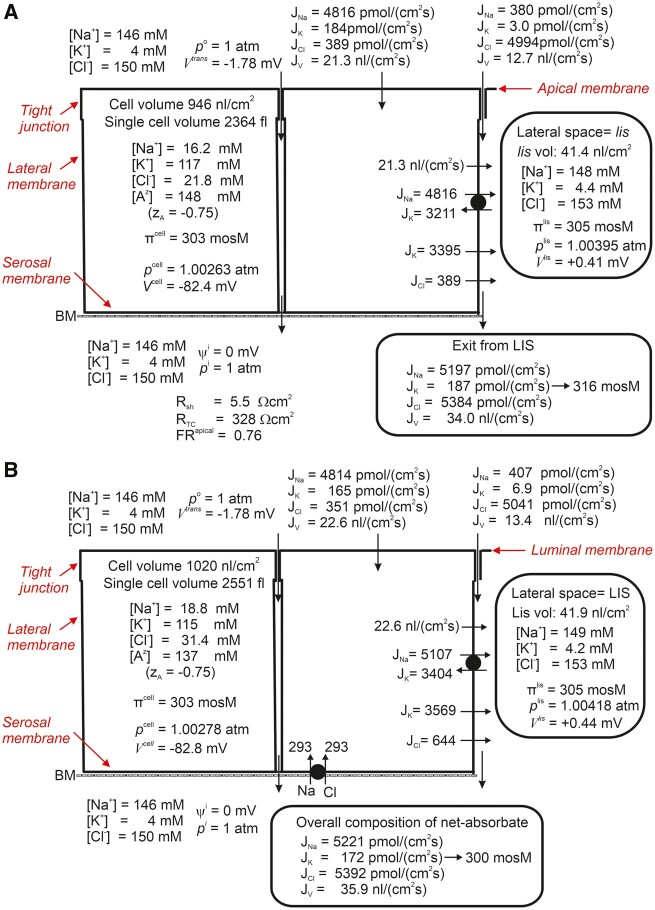
(**A**) With large experimental transepithelial flux of Na^+^ and hydraulic conductances, the fluid exiting the *lis* (through the basement membrane [BM]) is predicted to be hyperosmotic to the bath: 316 mosM versus 300 mosM. Individual plasma membranes are indicated in red color. Electrical resistances given by the model: *R^am^* = 313 Ω cm^2^, *R^lm^* = 16 Ω cm^2^, *R^tj^* = 4.4 Ω cm^2^, *R^ibm^* = 1.10 Ω cm^2^. FR^apical^ is the ratio of apical (luminal) membrane resistance to transcellular resistance.^3^ (**B**) Model solution illustrating how true isosmotic transport is obtained by recirculating Na^+^ from the serosal compartment into the lateral intercellular compartment (*lis*). All independent variables are similar to those used in computations of Figure 1A in which the 1Na^+^:1Cl^−^ cotransporter in serosal membrane is quiescent.^3^

Our study discloses a functionally important, but hitherto overlooked, instantaneous cross-talk between apical glucose uptake, lateral Na^+^ pump flux, and transepithelial water absorption. The sequence of mechanistic interactions is as follows: Adding glucose to the luminal fluid stimulates rheogenic glucose uptake that instantaneously depolarizes the cell, thus activating the voltage-dependent lateral Na^+^/K^+^ pump flux, which in turn energizes water uptake via the lateral intercellular coupling compartment.

It is a result of decisive significance for extracellular fluid homeostasis that the Na^+^ flux through the tight junctions is inward, driven by paracellular solvent drag, rather than being outward along the prevailing electrochemical potential gradient (Figure 1A). With glucose uptake, the junctional leak fluxes are similarly predicted to be effectively eliminated by solvent drag, which generates a paracellular Na^+^ flux of >600 pmol/(cm^2^s).^3^ Because this component bypasses the lateral Na^+^/K^+^ pump absorption of filtered fluid is energetically more efficient than predicted from the turnover of the lateral pump itself.

A simulated ouabain experiment revealed a neglected aspect of the importance of pump activity for the lateral membrane potential. Thus, a fast inhibition of the Na^+^/K^+^-ATPase, corresponding to a pump current inhibition of 155 µA/cm^2^ (one-third of the blocked active Na^+^ pump flux), generates an equally fast membrane depolarization of 40 mV. This follows from the pump being rheogenic with a turnover rate in the kidney ranking among the highest in nature.^6^ This is in contrast to the very small pump currents of excitable membranes which make a negligible contribution to the membrane potential.^7^

In a simulated apical-aquaporin knockout, we showed^3^ that all water uptake is redirected to the paracellular route. Halving the Na^+^ uptake leads to a 50% reduction in transepithelial water absorption quantitatively in agreement with experimental results from AQP-1 null mice.^8^ Generally, at transepithelial osmotic equilibrium, the hydraulic conductance of epithelial membranes directs the osmolality of absorbed fluid, while water uptake is determined solely by the rate at which Na^+^ is pumped into the *lis* no matter whether the water flux takes a paracellular or a transcellular route.^3^

We resolve the enigma of the hydraulic conductance of individual plasma membranes being larger by an order of magnitude compared to the hydraulic conductance of the intact tubule epithelium. With a constant compliance of gall bladder cells,^9^ computations given by the model demonstrate how a reversible stepwise increase in luminal osmolarity evokes a dual response of apical water flow $\left(J_{V}^{am}\right)$. An initial fast reversal of $J_{V}^{am}$ defined by the pulse amplitude is followed by a slow membrane compliance-dependent reversal of $J_{V}^{am}$ energized by the Na^+^/K^+^ pump. This time-dependent response rules out using the steady-state transepithelial water flux for calculating the transepithelial water permeability, which has no functional significance.

Our finding^3^ that the paracellular coupling of water flow and active Na^+^ flux inevitably results in a hyperosmotic absorbate shows that isosmotic transport is the result of regulation at the epithelial cell level. Following Hans Ussing,^10^ we introduce Na^+^ recirculation via a hypothetical Na^+^ cotransport mechanism in the serosal membrane. As indicated in Figure 1B, with this configuration of ion transporters true isosmotic transport is obtained by regulated Na^+^ recirculation amounting to a few percentages of the translateral Na^+^ uptake. Thus, the cost of producing isosmotic absorption by the highly water permeable kidney tubule is insignificant compared to small intestine of >50% regulated recirculation as indicated in experiment on toad small intestine^10^ and supported by subsequent computational analysis.^4^ The above prediction focuses future research on the nature and operation of serosal Na^+^ cotransporters which may have evolved for the purpose of securing Na^+^ recirculation and truly isosmotic transport in the different segments of proximal tubule.

## Funding

The study is supported by grants CF17-0186 and CF18-0661 from the Carlsberg Foundation.

## Conflicts of Interest Statement

None declared.
